# Development of a Reflective Electrochromic Zinc-Ion Battery Device for Infrared Emissivity Control Using Self-Doped Polyaniline Films

**DOI:** 10.3390/polym17152110

**Published:** 2025-07-31

**Authors:** Yi Wang, Ze Wang, Tong Feng, Jiandong Chen, Enkai Lin, An Xie

**Affiliations:** 1 Key Laboratory of Functional Materials and Applications of Fujian Province, School of Materials Science and Engineering, Xiamen University of Technology, Xiamen 361024, China; 2322161045@stu.xmut.edu.cn (Z.W.); cjd2975268317@163.com (J.C.); 19859769527@163.com (E.L.); anxie@xmut.edu.cn (A.X.); 2 National Key Laboratory of Electronic Thin Films and Integrated Devices, National Engineering Research, University of Electronic Science and Technology of China, Chengdu 610054, China; 3 School of Mechanical Electrical and Information Engineering, Xiamen Institute of Technology, Xiamen 361021, China

**Keywords:** electrochromism, infrared emissivity modulation, smart camouflage, multifunctional integration

## Abstract

Electrochromic devices (ECDs) capable of modulating both visible color and infrared (IR) emissivity are promising for applications in smart thermal camouflage and multifunctional displays. However, conventional transmissive ECDs suffer from limited IR modulation due to the low IR transmittance of transparent electrodes. Here, we report a reflection-type electrochromic zinc-ion battery (HWEC-ZIB) using a self-doped polyaniline nanorod film (SP(ANI-MA)) as the active layer. By positioning the active material at the device surface, this structure avoids interference from transparent electrodes and enables broadband and efficient IR emissivity tuning. To prevent electrolyte-induced IR absorption, a thermal lamination encapsulation method is employed. The optimized device achieves emissivity modulation ranges of 0.28 (3–5 μm) and 0.19 (8–14 μm), delivering excellent thermal camouflage performance. It also exhibits a visible color change from earthy yellow to deep green, suitable for various natural environments. In addition, the HWEC-ZIB shows a high areal capacity of 72.15 mAh cm^−2^ at 0.1 mA cm^−2^ and maintains 80% capacity after 5000 cycles, demonstrating outstanding electrochemical stability. This work offers a versatile device platform integrating IR stealth, visual camouflage, and energy storage, providing a promising solution for next-generation adaptive camouflage and defense-oriented electronics.

## 1. Introduction

Electrochromic devices possess the ability to modulate optical properties in the visible range, with this functionality extendable into the infrared region to enable dynamic control of surface thermal radiation [1,2]. By exploiting this feature, these devices can be integrated into advanced camouflage systems, wherein the infrared emissivity is adaptively tuned to match environmental radiative profiles, thus achieving efficient thermal concealment and visual integration [3,4]. By regulating both the visible color and the infrared emissivity, electrochromic devices overcome the limitations of conventional camouflage materials, whose color and infrared features are typically fixed and non-adjustable [5,6]. Therefore, the development of electrochromic devices with tunable color and infrared emissivity holds significant scientific and practical value [7].

Traditional transmissive sandwich-type electrochromic devices, typically fabricated with ITO or FTO electrodes, exhibit low transmittance in the infrared spectral region due to the surface electrodes, which adversely affects the modulation performance of the electrochromic active materials, thereby limiting the realization of effective infrared regulation [8]. The key performance metric for infrared-regulating electrochromic devices is the emissivity modulation range, which is strongly influenced by the surface state of the device’s active layer [9]. Consequently, reflective-type electrochromic device architectures offer a distinct advantage for achieving infrared emissivity modulation. Positioning the electrochromic active layer at the outermost surface of the device enables reflective configurations to modulate infrared emissivity through direct control of the surface state. This approach effectively mitigates the limitations associated with emissivity regulation in traditional structures that employ conductive glass electrodes [10,11].

To date, significant progress has been made worldwide in the development of infrared-regulating electrochromic devices [12,13,14]. Zhang et al. [15] fabricated CSA-doped polyaniline (PANI) films on Au-coated porous nylon 66 substrates via electrochemical deposition. The resulting films exhibited an emissivity modulation of 0.225 in the 3–5 μm infrared range. Wang et al. [16] fabricated a symmetric electro-emissive device using a biomimetic PANI/Ce^4+^ film, achieving an IR emissivity modulation of 0.58 in the 8–14 μm range with excellent flexibility and cycling stability. Zhao et al. [17] developed a flexible multilayer graphene-based infrared device on a porous polyethylene membrane, where the emissivity was tunable from 0.57 to 0.41 via ionic liquid intercalation. Zhang et al. [2] developed a flexible WO_3_-based electrochromic device exhibiting reversible color changes from yellow to dark green, with a reflectance modulation of 38.9% across the 250–2500 nm wavelength range. Most reported electrochromic devices with mid- to far-infrared modulation capabilities primarily adopt a symmetric supercapacitor structure, which typically consists of two identical electrodes assembled in a sandwich configuration. Most reported electrochromic devices capable of mid- to far-infrared modulation adopt a symmetric supercapacitor-like configuration, in which two identical electrodes are assembled into a sandwich structure [18]. However, the overall performance of such devices is often constrained by the inferior electrode, and the architecture requires a high degree of electrochemical and optical compatibility between both electrodes. In contrast, replacing one electrode with a metal foil to form a “battery-type” device, similar to a secondary metal-ion battery, not only simplifies fabrication but also significantly reduces active material usage. In our previous work, a reflective thin-film device featuring a “battery-type” structure was designed, employing polyaniline (PANI) as the electrochromic active material and metallic zinc as the anode. This device demonstrated excellent visible-light color modulation coupled with superior energy storage performance [19]. Extending the modulation wavelength range of such “battery-type” reflective thin-film devices to encompass infrared regulation, and developing electrochromic–battery hybrid devices with infrared emissivity control, presents promising opportunities for broader integration of electrochromic functionalities with secondary ion battery systems [20]. Notably, infrared-regulating electrochromic devices based on this “battery-type” configuration remain scarcely reported, underscoring the novelty and significance of further exploration in this domain.

In this work, PANI films with self-doping sulfonic acid groups on the polymer chains, denoted as SP(ANI-MA), were synthesized via the electrochemical copolymerization of aniline monomer and 3-aminobenzenesulfonic acid. Subsequently, the SP(ANI-MA) film was used as the cathode and zinc foil as the anode to assemble an infrared electrochromic zinc-ion battery device (HWEC-ZIB). Considering the significant influence of surface properties on the infrared emissivity modulation performance, various transparent encapsulation films were screened to optimize the device surface. Additionally, the effect of the electrolyte on infrared regulation was systematically investigated, and the modulation mechanisms of the materials were elucidated. Further studies on the HWEC-ZIB device demonstrated its multifunctional characteristics, exhibiting excellent electrochromic performance in both visible and infrared spectra, along with promising energy storage capabilities. These findings indicate that the developed device holds considerable potential for applications in future intelligent camouflage technologies.

## 2. Experimental Section

### 2.1. Materials

Aniline, 3-aminobenzenesulfonic acid, zinc chloride (ZnCl_2_, 98%), poly(methyl methacrylate) (PMMA), and propylene carbonate (PC) were purchased from Aladdin Reagent Co., Ltd. (Shanghai, China). Zinc foil was supplied by Zhongyan Metal Materials (Zhengzhou, China). The polyethylene (PE) film used for encapsulation was made of linear low-density polyethylene (LLDPE) with a thickness of approximately 20 μm. Perchloric acid (HClO_4_) and sulfuric acid (H_2_SO_4_) were obtained from Kelong Chemical Reagents Co., Ltd. (Chengdu, China). Flexible Au/Nylon 66 composite electrodes were fabricated based on our previously established protocols [19,21].

### 2.2. Fabrication of SP(ANI-MA) Electrodes

The electrodeposition was conducted in an aqueous solution of 1 mol L^−1^ perchloric acid (HClO_4_), containing a total monomer concentration of 0.1 mol L^−1^. An equimolar ratio (1:1) of aniline and 3-aminobenzenesulfonic acid was used as the monomer mixture. A three-electrode configuration was employed during deposition, where the working electrode consisted of a Au-coated porous Nylon 66 membrane (Au/Nylon 66). The preparation of the Au/Nylon 66 porous electrode follows the method reported in our previous publication [19]. A platinum foil served as the counter electrode, and a Ag/AgCl electrode was used as the reference. Electropolymerization was carried out at a constant current density of 0.25 mA cm^−2^ for a total deposition time of 7200 s.

### 2.3. Preparation of Gel Electrolytes

A total of 1 g of poly(methyl methacrylate) (PMMA) was dispersed in 5 g of propylene carbonate (PC) within a round-bottom flask. Subsequently, 5 mL of acetonitrile, 0.4 g of lithium perchlorate (LiClO_4_), and 0.4 g of zinc chloride (ZnCl_2_) were introduced. The mixture was then stirred and gradually heated to 90 °C until a clear and uniform gel electrolyte was obtained.

### 2.4. Assembly Procedure of Electrochromic Film Devices

**Scheme 1:**
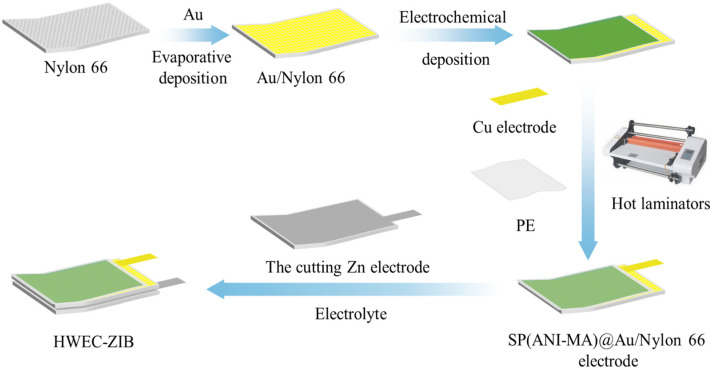
Schematic illustration of the encapsulation process for an HWEC-ZIB.

The surface condition of electrochromic films plays a crucial role in determining their infrared modulation performance. To enhance the IR regulation capability, surface encapsulation is essential, as it not only protects the electroactive materials but also mitigates electrolyte infiltration that may interfere with the modulation effectiveness. Therefore, effective encapsulation of the infrared electrochromic device plays a crucial role in ensuring its performance and stability. As illustrated in Scheme 1, a portion of the SP(ANI-MA) film is intentionally left uncoated to serve as the contact area. A highly conductive copper foil is used to lead out the electrode, thereby avoiding repeated clamping directly on the Au/Nylon 66 surface during measurement, which could otherwise damage the conductive layer. A PE film is carefully laid flat over the electrochromic surface of the SP(ANI-MA) film. The assembly is then sandwiched between smooth aluminum foils and thermally laminated at 125 °C using a roller laminator. After cooling, the laminated film is placed flat on a clean surface, and the gel electrolyte is applied to the non-Au-coated side. The sample is then subjected to repeated vacuum cycles in a vacuum chamber, promoting effective penetration of the electrolyte into the porous structure of the Nylon 66 substrate. A pre-cut zinc foil is then placed in contact with the gel-coated side of the film, forming a bilayer electrode structure. The back side is sealed with PE film, and the edges are thermally laminated using a heat-press sealer to prevent electrolyte leakage. For comparison, control devices were assembled without surface thermal lamination. The electrolyte was uniformly spread over the surface of the SP(ANI-MA) film, and a PE film was subsequently layered on top. Excess electrolyte was gently pressed out, and the subsequent assembly steps were carried out following the same procedure as that used for the laminated devices.

### 2.5. Characterization

The microstructural features of the Au/Nylon 66 substrates and the SP(ANI-MA)-based electrochromic films were characterized by field-emission scanning electron microscopy (FE-SEM, GeminiSEM 300, Carl Zeiss, Oberkochen, Germany), in conjunction with energy-dispersive X-ray spectroscopy (EDS, Smartedx, Oxford Instruments, Oxford, UK) for elemental mapping and analysis. The chemical composition and surface elemental states were further examined using X-ray photoelectron spectroscopy (XPS, Kratos XSAM800, Kratos Analytical Ltd., Manchester, UK). The reflectance spectra of the electrochromic layers, covering the ultraviolet to near-infrared region, were collected using a PerkinElmer Frontier FT-IR spectrometer (PerkinElmer Inc., Waltham, MA, USA). The electrochemical performance of the HWEC-ZIB devices was evaluated using cyclic voltammetry (CV), galvanostatic charge–discharge (GCD), and chronoamperometry techniques. CV measurements were conducted within a voltage range of 0.5–1.6 V at various scan rates from 2 to 50 mV s^−1^. GCD tests were performed in the same voltage window (0.5–1.6 V) with current densities ranging from 0.1 to 5 mA cm^−2^. The electrochromic switching behavior was analyzed by applying step voltages of 1.6 V (coloring) and 0.5 V (bleaching) with a step interval of 30 s, during which the current–time (I–t) responses were recorded to determine the coloration and bleaching response times. All electrochemical tests were carried out at room temperature under ambient conditions.

## 3. Results and Discussion

### 3.1. Characterization and Analysis of the Surface Encapsulation Layer and SP(ANI-MA) Active Material in HWEC-ZIB

**Figure 1:**
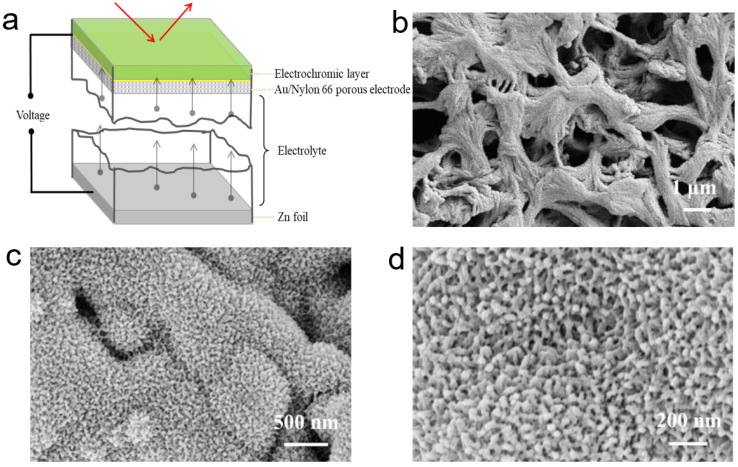
(**a**) Structure of the infrared-regulating device; (**b**) SEM image of the Au/Nylon 66 flexible electrode surface; (**c**,**d**) SEM images of the SP(ANI-MA) film surface morphology.

In the reflective infrared modulation device architecture, ensuring the electrochromic active layer’s infrared modulation performance requires minimizing the impact of external factors such as surface encapsulation materials and electrolyte infiltration on its modulation capability. The device structure employed in this work is depicted in Figure 1a. The electrochromic layer is encapsulated beneath an infrared-transparent film at the outermost surface of the device, while the electrolyte is confined within the porous conductive electrode substrate. Metallic zinc foil serves as the device’s counter electrode. The surface encapsulation film is crucial for practical applications, but to ensure it does not impair infrared modulation performance, it must have high infrared transmittance. Moreover, the encapsulation material should effectively inhibit electrolyte permeation to the surface layer to avoid deterioration of the infrared modulation capability caused by electrolyte absorption. The transmittance performance of the encapsulation films is primarily evaluated in the 3~5 μm and 8~14 μm wavelength ranges. Transmission spectra of different film samples, including polyethylene (PE), polypropylene (PP), and polyethylene terephthalate (PET) membranes, are presented in Figure S1a–c. In the 3~5 μm band, the PE film shows comparable transmittance to PP and PET films, while the polytetrafluoroethylene (PTFE) film exhibits significantly higher transmittance than the other three materials. Within the 8–14 μm range, the average reflectance of PE film is markedly higher than that of PP, PET, and PTFE films. The relatively simple molecular structure of PE results in minimal absorption or vibrational effects in the infrared region, especially in the 3~5 μm band. Therefore, PE film was selected as the surface encapsulation layer material for device packaging to optimize infrared modulation performance.

The morphology of the synthesized SP(ANI-MA) film and the Au/Nylon 66 electrode was examined using scanning electron microscopy (SEM). As shown in Figure 1b, the surface of the Au/Nylon 66 electrode exhibits a cross-linked fibrous structure with abundant porous features. The morphology of the SP(ANI-MA) film deposited on the Au/Nylon 66 substrate is displayed in Figure 1c,d. The SP(ANI-MA) material is uniformly deposited on the porous fibrous network, forming densely packed, short nanorod-like structures. The resulting film demonstrates a highly porous morphology with a large specific surface area, which facilitates enhanced contact between the electrolyte and the active material, thereby promoting improved electrochemical performance. To further confirm the elemental composition and distribution, energy-dispersive X-ray spectroscopy (EDS) mapping was performed on the SP(ANI-MA) film, as shown in Figure S2. The characteristic elements of the film, including C, N, O, S, and Cl, are uniformly distributed throughout the structure, indicating the homogeneity of the synthesized SP(ANI-MA) film. The sulfur element originates from the 3-aminobenzenesulfonic acid monomer, while the chlorine element comes from perchlorate ions introduced by the deposition electrolyte. The presence of chlorine indicates that perchlorate anions are incorporated into the polymer chains during the electrochemical polymerization process, likely serving as counterions for doping.

### 3.2. Study on the Infrared Emissivity Modulation Performance of Electrochromic Film Devices

The tunable range of infrared emissivity is one of the most critical parameters for evaluating the infrared regulation performance of a device. When incident radiation strikes the surface of an object, the sum of its reflectance (*R*), absorptance (*A*), and transmittance (*T*) satisfies the energy conservation relationship:

*R + A + T* = 1 (1)

According to Kirchhoff’s law of thermal radiation, the emissivity (*ε*) of a surface at thermal equilibrium is equal in magnitude to its absorptance at the same temperature. Given that the device developed in this work adopts a reflective structure, the transmittance can be regarded as negligible (*T* ≈ 0), and Equation (1) can be simplified to describe the relationship between reflectance and emissivity as follows [10,22]:

*R + ε* = 1 (2)

**Figure 2:**
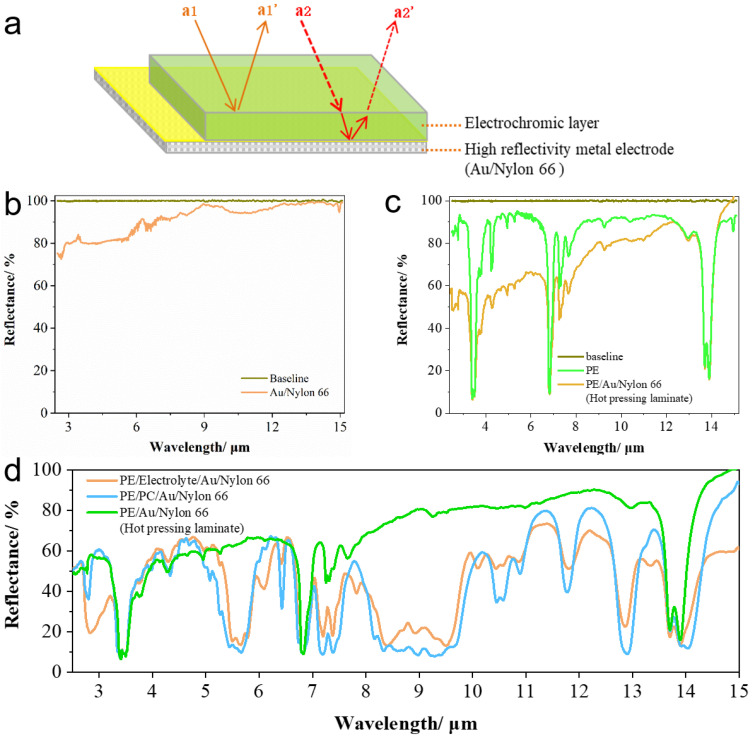
(**a**) Schematic illustration of the modulation mechanism of the PANI-based reflective infrared electrochromic device; (**b**) reflectance spectrum of the Au/Nylon 66 flexible electrode, highlighting its broadband reflective properties in the infrared region; (**c**) comparison of the reflectance spectra of a PE film alone and the same PE film thermally laminated onto the Au/Nylon 66 flexible electrode, demonstrating the effect of lamination on infrared optical behavior. (**d**) Reflectance spectra of different samples.

In this study, the infrared reflectance spectra of the device were measured using a Fourier-transform infrared (FTIR) spectrometer. The instrument’s built-in pure gold foil served as the calibration standard during measurements. Currently, the widely accepted mechanism for reflectance modulation in PANI-based electrochromic devices suggests that under an applied electric field, the transmittance of the electrochromic material changes significantly, whereas the intrinsic reflectivity of the material (a1’) remains relatively constant. The observed modulation in overall reflectance arises primarily from the change in transmittance, which in turn alters the intensity of the reflected light from the underlying reflective substrate (a2’). A schematic illustration of this modulation mechanism is shown in Figure 2a.

Recent studies have demonstrated that the modulation of infrared emissivity in PANI-based electrochromic devices is primarily governed by the redox-induced formation and annihilation of charge carriers—specifically, polarons and bipolarons—on the PANI polymer backbone [15,23,24]. In the reduced (de-doped) state, where PANI lacks both polarons and bipolarons, the polymer exhibits low electrical conductivity and high optical transparency in the infrared region. Under these conditions, the incident infrared radiation (a2) can effectively penetrate the PANI layer and be reflected by the highly reflective Au/Nylon 66 substrate beneath, resulting in a high total reflectance of the device—corresponding to a low-emissivity state. During oxidation (doping), a high density of bipolarons is generated along the polymer chains, imparting semiconducting or conductive behavior to the PANI layer. The increased carrier concentration enhances the infrared absorption of the PANI film and decreases its infrared transmittance, thereby significantly suppressing the reflected component and leading the device to exhibit a low-reflectance (high-emissivity) state. By controlling the reversible redox state of the PANI layer, the infrared electrochromic device can effectively modulate its infrared emissivity [23]. The reflectance spectrum of the Au/Nylon 66 flexible electrode was measured, as shown in Figure 2b. The electrode exhibited a consistently high reflectance across a broad spectral range. Based on the reflectance data, the infrared emissivity of the Au/Nylon 66 electrode was calculated. The results indicate an emissivity of 0.193 in the 3–5 μm band and 0.035 in the 8–14 μm band, confirming the intrinsically low-emissivity nature of the Au/Nylon 66 substrate in both mid-wave and long-wave infrared regions. To minimize external interference and preserve the emissivity-modulating capability of the active electrochromic layer, a polyethylene (PE) film with high infrared transmittance was selected as the surface encapsulation layer. The reflectance characteristics of the PE film were investigated and are presented in Figure 2c. The film demonstrated relatively high reflectance, which corresponds to good transmittance in the infrared region. However, certain dips in reflectance were observed in specific spectral windows—namely 3.3–3.5 μm, 5.1–5.2 μm, and 13.3–14.2 μm. These localized decreases in reflectance are attributed to intrinsic absorption bands of the PE material, which attenuate transmission within these regions [25].

To further assess the effect of the PE encapsulation film on reflectance, a PE film was thermally laminated onto the surface of the Au/Nylon 66 flexible electrode, and its reflectance spectrum was recorded (Figure 2c). The results show a marked decrease in reflectance within the 3–5 μm range and a slight reduction in the 8–14 μm range, indicating that the laminated PE film slightly reduces the overall reflectance of the Au/Nylon 66 electrode. To assess the potential impact of electrolyte infiltration on infrared emissivity modulation, a small amount of electrolyte was applied to the surface of the Au/Nylon 66 substrate, and a PE film was subsequently placed on top. The electrolyte was gently compressed to minimize its presence at the measurement interface, simulating a scenario of partial surface infiltration. The reflectance spectrum of the resulting multilayer structure is shown in Figure 2d. The results indicate a significant reduction in reflectance, with multiple disordered reflection peaks observed. The calculated infrared emissivity increased to 0.41 in the 3–5 μm range and 0.56 in the 8–14 μm range, reflecting a substantial loss of the original low-emissivity characteristics of the Au/Nylon 66 electrode. To identify the component responsible for the disturbed reflectance behavior, a control sample was prepared by applying a small amount of propylene carbonate (PC)—a common electrolyte solvent—between the PE film and the Au/Nylon 66 substrate. The reflectance spectrum of this sample showed a highly similar pattern to that of the PE/electrolyte/Au/Nylon 66 configuration, suggesting that the observed optical degradation may primarily originate from PC solvent absorption in the infrared region. These findings highlight the importance of preventing electrolyte infiltration into the surface layer, as even minor penetration of solvent components can substantially disrupt the emissivity modulation performance of the device.

**Figure 3:**
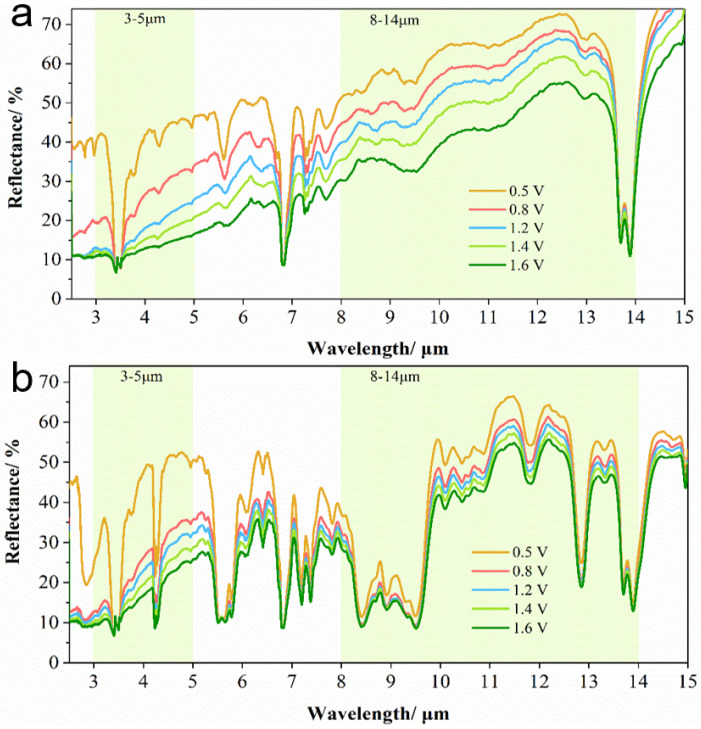
Infrared reflectance spectra of the devices: (**a**) HWEC-ZIB device; (**b**) HWEC-ZIB(Electrolyte) device.

To further evaluate the infrared modulation performance of the prepared SP(ANI-MA)-based electrochromic films, devices were assembled using thermally laminated encapsulation and tested under various operating conditions. For clarity, the device encapsulated with a PE film via thermal lamination is denoted as HWEC-ZIB, while the device assembled by simply drop-casting electrolyte onto the surface followed by covering with a PE film (without thermal sealing) is denoted as HWEC-ZIB(Electrolyte). Figure 3a displays the infrared reflectance spectra of the HWEC-ZIB device in the 2.5–15 μm wavelength range under various applied voltages. When a voltage of 0.5 V is applied—corresponding to the reduced state of the SP(ANI-MA) film—the polymer backbone remains in a non-conductive state, with no formation of polarons or bipolarons. In this state, the film exhibits a high infrared transmittance, allowing incident IR radiation to pass through the SP(ANI-MA) layer and be efficiently reflected by the underlying Au/Nylon 66 electrode. This results in an overall high reflectance (low emissivity) state of the device [26]. As the applied voltage increases, the SP(ANI-MA) film undergoes oxidation and enters the doped state, accompanied by the generation of polarons and bipolarons along the polymer chains. This leads to increased infrared absorption and a corresponding decrease in transmittance. Consequently, less infrared radiation reaches the reflective Au/Nylon 66 electrode and is reflected back, resulting in a gradual decline in reflectance and an increase in emissivity [27]. Once the voltage exceeds 1.6 V, further increases produce negligible changes in reflectance, indicating a saturation of the doping process.

The infrared emissivity modulation range under different applied voltages was calculated based on the reflectance spectra, and the results are summarized in Table 1. For the HWEC-ZIB device, the modulation range reaches 0.28 in the 3–5 μm region and 0.19 in the 8–14 μm region, demonstrating effective infrared tunability. To further investigate the influence of electrolyte exposure, the infrared reflectance spectra of the HWEC-ZIB(Electrolyte) device were measured, as shown in Figure 3b. In this configuration, the electrolyte is directly applied onto the surface of the SP(ANI-MA) film without thermal sealing. The components of the electrolyte exhibit inherent absorption in the mid-infrared region, resulting in the presence of more numerous and irregular low-reflectance peaks compared to the HWEC-ZIB device. The emissivity values under maximum modulation conditions for both devices are listed in Table 1. Compared to HWEC-ZIB, the HWEC-ZIB(Electrolyte) device exhibits poorer modulation performance in the 8–14 μm range, with a modulation range of only 0.09, and a slightly reduced range of 0.26 in the 3–5 μm region. These results highlight that the presence of electrolyte at the device surface significantly compromises infrared reflectance, likely due to the absorption contribution from electrolyte components such as PC solvent. Based on the infrared modulation mechanism of the material and the observed degradation caused by surface electrolyte infiltration, it can be concluded that electrolyte penetration into the active surface of the device should be avoided to maintain optimal infrared modulation performance. In contrast, devices encapsulated via thermal lamination with PE films demonstrate superior emissivity modulation capability, underscoring the importance of proper device packaging in achieving effective mid-infrared tunability. The performance comparison of infrared modulation electrochromic devices is summarized in Table 2.

### 3.3. Electrochemical Energy Storage and Electrochromic Performance of the Film Device

**Figure 4:**
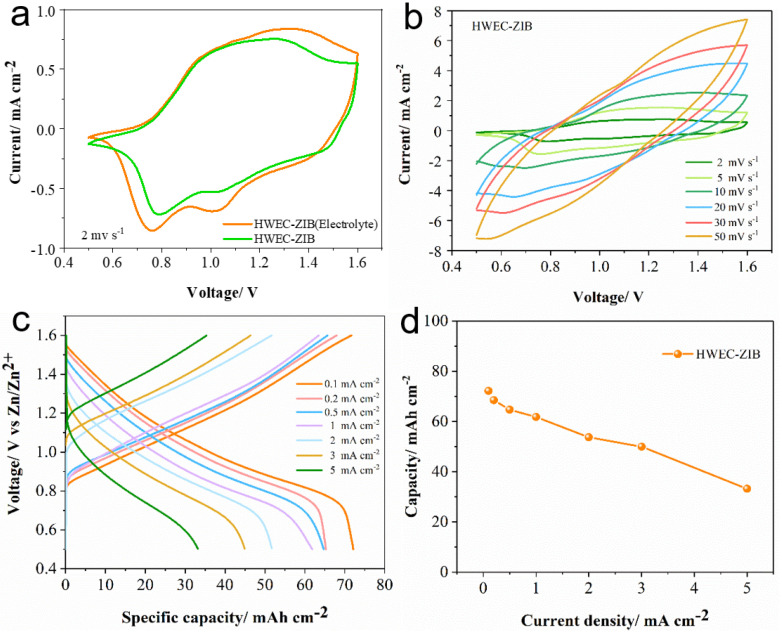
Electrochemical energy storage performance of the devices. (**a**) CV curves at a scan rate of 2 mV s^−1^; (**b**) CV curves of the HWEC-ZIB device at different voltages; (**c**) GCD curves of the HWEC-ZIB device; (**d**) Capacity statistics of the HWEC-ZIB device.

The cyclic voltammetry (CV) profiles of the fabricated devices, recorded within the voltage window of 0.5–1.6 V, are presented in Figure 4a. Multiple well-defined redox couples are observed, corresponding to the reversible redox transitions of the SP(ANI-MA) layer at different potentials. A comparison of the CV curves for HWEC-ZIB and HWEC-ZIB(Electrolyte) reveals that the enclosed area—and hence the charge-storage capability—of the encapsulated HWEC-ZIB device is smaller than that of its non-sealed counterpart. This reduction is attributed to the intimate contact between the thermally laminated PE film and the SP(ANI-MA) surface, which partially shields the active area and consequently diminishes the electrochemical response. As the scan rate increases, the CV curves of the HWEC-ZIB device (Figure 4b) gradually exhibit a more spindle-like shape, indicative of increased electrochemical polarization. Such behavior implies elevated internal resistance, possibly caused by structural densification or microcrack formation during hot-press lamination, both of which can obstruct ionic and electronic transport pathways within the film. Galvanostatic charge–discharge (GCD) measurements were employed to quantify the areal capacity (Figure 4c). The HWEC-ZIB device delivers an areal capacity of 72.15 mAh cm^−2^ at 0.1 mA cm^−2^ and 61.8 mAh cm^−2^ at 1 mA cm^−2^. Figure 4d presents the capacity retention across varying current densities. When the current density is increased by 50-fold, the device still retains 46% of its initial capacity, demonstrating a satisfactory rate performance.

**Figure 5:**
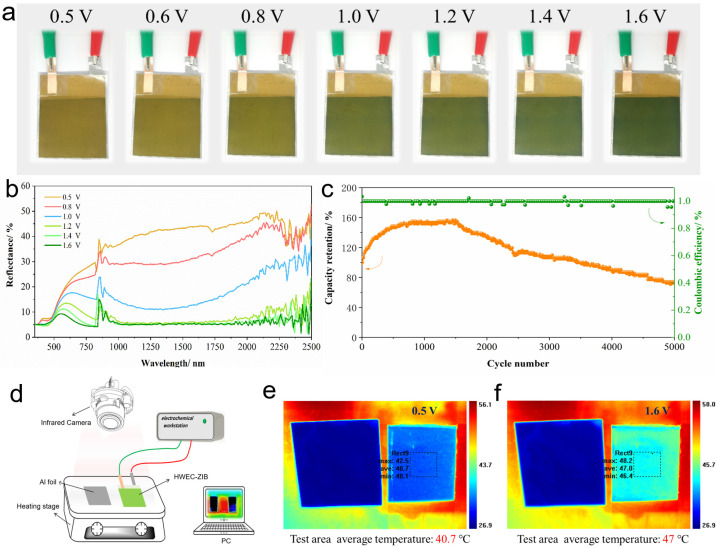
(**a**) Photographs of the HWEC-ZIB under different applied voltages, showing visible color transitions corresponding to electrochemical states; (**b**) UV–Vis–NIR spectral response of the HWEC-ZIB device at various voltages, indicating its voltage-dependent optical modulation capability; (**c**) stability test curves of the HWEC-ZIB device; (**d**) schematic illustration of the infrared regulation performance testing setup; (**e**) thermal images of the HWEC-ZIB device at different voltages; (**f**) thermal images of the HWEC-ZIB device under various voltages.

The HWEC-ZIB device, which exhibited notable infrared emissivity modulation, was further evaluated for its visible light modulation performance by capturing optical images under different applied voltages. As shown in Figure 5a, within the voltage range of 0.5–1.6 V, the device undergoes a distinct color change from light yellow (fully reduced state) to dark green (fully oxidized state), indicating a strong visible light electrochromic response. To further elucidate the device’s optical modulation capabilities across a broad spectral range, reflectance spectra were measured in the 350–2500 nm wavelength range under different applied voltages, as shown in Figure 5b. At 0.5 V, the film exhibits a yellow color with the highest reflectance, and the average reflectance in the 1000–2500 nm near-infrared region is approximately 40%. As the voltage increases and the device transitions to green and eventually dark green, the reflectance continuously decreases. At 1.6 V, the average reflectance drops sharply to around 5% over the same spectral range. These results demonstrate that the HWEC-ZIB device exhibits excellent optical modulation not only in the visible region but also in the near-infrared region, highlighting its potential for applications requiring broadband electro-optical control.

The response times of the fabricated HWEC-ZIB device were evaluated by recording the current–time profiles under applied coloration and bleaching potentials. The coloration voltage was set to 1.6 V, and the bleaching voltage to 0.5 V, with a voltage step duration of 30 s, as presented in Figure S3. The calculated coloration and bleaching response times for the SP(ANI-MA)-based infrared electrochromic zinc-ion battery device were approximately 13.3 s and 9.25 s, respectively. The relatively slow response speed can be attributed to the diminished electrochemical activity of the electrochromic film after thermal compression encapsulation. The encapsulation process may induce morphological changes or restrict ion transport within the active layer, thereby hindering the electrochromic switching kinetics.

The cycling stability of the HWEC-ZIB device was further evaluated, as shown in Figure 5c. During the initial ~1300 cycles, the device exhibited a gradual increase in storage capacity, likely attributed to an activation process where the porous structure within the film becomes more accessible, facilitating enhanced ion doping of the electrolyte [28]. With continued cycling, a gradual decline in performance was observed, which can be ascribed to the repeated doping and dedoping of electrolyte ions causing partial collapse and degradation of the polymer chain segments, thereby reducing the electrochemical activity. Nevertheless, after 5000 cycles, the device retained approximately 80% of its initial capacity, and the Coulombic efficiency remained stable, demonstrating excellent cycling durability and electrochemical stability.

### 3.4. Investigation of Infrared Emissivity Modulation Performance and Applications of Film Devices

The infrared emissivity modulation performance of the film device can be visually demonstrated using an infrared (IR) camera combined with auxiliary software to capture the IR thermal images of the device surface. The testing setup is illustrated in Figure 5d. During testing, the device is secured onto a heated stage, with thermal grease applied between the film and the stage to improve heat transfer, ensuring the device surface temperature closely matches that of the stage. By applying a voltage to the device, the surface emissivity can be precisely controlled. When switched to a high-emissivity state, the film’s thermal radiation output increases significantly, resulting in a noticeably higher apparent temperature captured by the IR camera. Conversely, when switched to a low emissivity state, the thermal radiation from the surface decreases, and the IR camera shows a lower surface temperature. The emissivity modulation effect can be intuitively observed by monitoring the color changes and temperature readings of the target area within the IR camera software interface. For clearer comparison, an aluminum foil sample with low emissivity was used as a reference, appearing as deep blue on the infrared camera display. When the device voltage is set to 0.5 V, the film is in the dedoped state, exhibiting low emissivity and correspondingly low thermal radiation emission, as shown in the IR image in Figure 5e (left). When the voltage is increased to 1.6 V, the film is doped, showing higher emissivity and increased thermal radiation emission, as shown in Figure 5f. By analyzing the average temperature within the designated area on the IR camera software, it is found that at a background temperature (heated stage temperature) of approximately 55 °C, the fabricated device demonstrates a temperature modulation capacity of about 6.3 °C. This indicates that the HWEC-ZIB device possesses effective emissivity regulation ability adaptable to varying environmental temperatures, highlighting its potential application in thermal infrared camouflage technologies [29].

**Figure 6:**
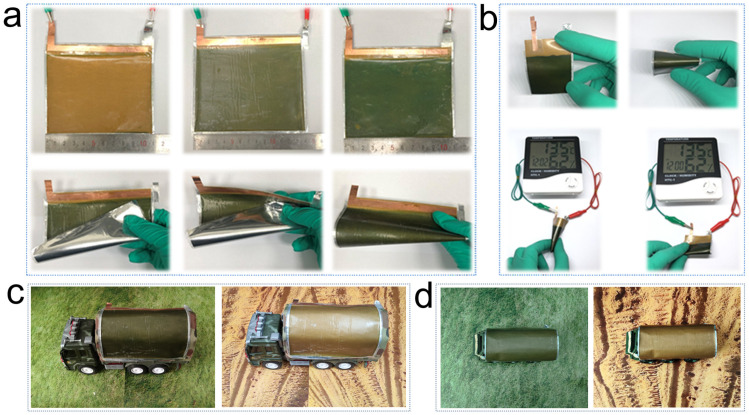
(**a**) Large-area device and its bending states; (**b**) small-area device driving an electronic display; (**c**,**d**) camouflage performance on a transport vehicle model.

Furthermore, large-area film devices with a size of 9 × 11 cm^2^ were successfully fabricated, as shown in Figure 6a, exhibiting excellent electrochromic performance. The devices were assembled using highly flexible Au/Nylon 66 electrodes coated with self-doped SP(ANI-MA) films as the positive electrode, paired with flexible and stable zinc foil as the negative electrode. As a result, the devices demonstrate outstanding mechanical flexibility; as illustrated, the film devices can undergo large-angle bending on any surface without damage. Additionally, due to their inherent energy storage capability, the fabricated small-area devices (2.5 × 3 cm^2^), shown in Figure 6b, are capable of powering a temperature and humidity meter. The device maintains stable operation even under different bending angles, indicating excellent mechanical robustness and functional reliability.

Moreover, the fabricated film devices exhibit a distinct color change from yellowish-brown to green in the visible spectrum, closely matching typical soil and grassland backgrounds. This characteristic endows the devices with significant potential for camouflage applications. To evaluate their suitability for camouflage, a transport vehicle model was used as the target, and the HWEC-ZIB device served as the camouflage film. Optical photographs were taken against grass and soil backgrounds for validation. As shown in Figure 6c,d, the fixed-pattern camouflage on the vehicle blends well with the grassland background but performs poorly against the soil background. In contrast, the HWEC-ZIB device, with its color-tunable property, demonstrates effective camouflage on both grass and soil backgrounds. This preliminary test confirms the film’s potential for camouflage use. In future applications, the film can be further patterned into arrays to adapt to a wider range of scenarios. Considering the HWEC-ZIB device’s combined capabilities in infrared emissivity modulation, visible light camouflage, and energy storage, this multifunctional film device holds promising prospects for advanced camouflage applications.

**Table 2:** Performance comparison of infrared modulation electrochromic devices.

Device Structure	Active Material	Wavelength Range (μm)IR Modulation Range (Δε)	Response Time (s)Tb/Tc	Energy Storage Capacity (mAh/cm^2^)	Reference
SP(ANI-MA)//Zn	Self-doped PANI (SP(ANI-MA))	0.28 @3–5 μm 0.19 @8–14 μm	9.25 s/13.3 s	72.15 @ 0.1 mA/cm^2^	This work
WO_3_//WO_3_	WO_3_/Au/nylon 66 porous	Reflectance modulation of 38.9% @250–2500 nm	~0.32 s	/	[2]
DBSA-PANI//DBSA-PANI	DBSA-doped PANI films	0.43 @8–14 μm 0.4 @2.5–25 μm	/	/	[30]
Reflective-type Zn-ions electrochromic devices WO_3_//Zn	WO_3_	0.26 @3–5 μm 0.31 @8–14 μm	7.2 s/11.7 s	/	[20]
TENG for building self-powered infrared detector	PANI with a sulfuric acid dopant	Average infrared reflectance contrast of 46% @8–14 μm	/	/	[31]
CSA-doped//CSA-PANI	CSA doped-PANI film	0.225 @3–5 μm 0.399 @8–12 μm 0.426 @2.5–25 μm	2.5 s/6 s	/	[15]
H_2_SO_4_-doped PANI//H_2_SO_4_-doped-PANI	H_2_SO_4_-doped PANI EC films	0.4 @8–14 μm 0.3 @2.5–25 μm	/	/	[27]
PANI/Au//PANI/Au	PANI/Au composite film	0.402 @8–14 μm	/	/	[32]
PANI//MXene/PANI/PVDF	PANI	0.39 @8–14 μm	/	/	[33]

Table footnotes: Tb: Bleaching response time; Tc: Coloration response time. DBSA: Dodecyl benzene sulfonic acid; TENG: Triboelectric nanogenerator. CSA: Camphorsulfonic acid; PVDF: Polyvinylidene fluoride.

## 4. Conclusions

In this work, a self-doped polyaniline-based film (SP(ANI-MA)) with nanorod morphology was synthesized via electrochemical copolymerization and integrated into a reflective HWEC-ZIB device. Systematic evaluation revealed that electrolyte infiltration impairs infrared modulation by increasing IR absorption. The optimized heat-pressed and sealed device achieved infrared emissivity modulation ranges of 0.28 (3–5 μm) and 0.19 (8–14 μm), demonstrating strong potential for thermal camouflage. The device also exhibited visible color tunability from yellowish-brown to deep green, effectively simulating natural backgrounds for visual concealment. Electrochemically, it delivered a stable areal capacity of 72.15 mAh cm^−2^ at 0.1 mA cm^−2^, maintaining 80% capacity retention after 5000 cycles, confirming its multifunctional capabilities for integrated infrared modulation, visible camouflage, and energy storage applications. To further enhance the infrared modulation range and overall device performance, future research could focus on tailoring the nanostructure and doping characteristics of the SP(ANI-MA) film to improve ionic and electronic transport kinetics. In addition, employing high-conductivity electrolytes and introducing advanced surface engineering strategies may offer effective routes to achieve broader and more stable emissivity modulation.

## Figures and Tables

**Table 1:** Infrared emissivity statistics of HWEC-ZIB-based electrochromic devices under different states.

Electrochromic Device	Emissivity	Infrared Wavelength Range		
		3~5 μm	8~14 μm	2.5~15 μm
HWEC-ZIB	Low emissivity of the device	0.58	0.39	0.43
	High emissivity of the device	0.86	0.59	0.62
	Modulation range (Δε)	0.28	0.20	0.19
HWEC-ZIB(Electrolyte)	Low emissivity of the device	0.54	0.57	0.58
	High emissivity of the device	0.80	0.66	0.67
	Modulation range (Δε)	0.26	0.09	0.09

## Data Availability

The original contributions presented in this study are included in the article. Further inquiries can be directed to the corresponding author.

## Supplementary Materials

The following supporting information can be downloaded at: https://www.mdpi.com/article/10.3390/polym17152110/s1, Figure S1. Comparison of infrared reflectance spectra of PE film with (a) PP, (b) PTFE, and (c) PET films. Figure S2. EDS mapping characterization of the SP(ANI-MA) film surface. Figure S3. Response time characterization curves of the HWEC-ZIB device.
