# Supplementary material for: Development of a Reflective Electrochromic Zinc-Ion Battery Device for Infrared Emissivity Control Using Self-Doped Polyaniline Films
Source: Polymers (Basel). 2025 Jul 31;17(15):2110. doi: 10.3390/polym17152110 (PMC12349100; doi:10.3390/polym17152110)
Supplement: Supplementary file 1 [file polymers-17-02110-s001.zip › polymers-3768659-supplementary.pdf]

Supporting Information

Development of a Reflective Electrochromic Zinc-Ion Battery Device for Infrared Emissivity Control Using Self-Doped Polyaniline Films

Yi Wang[1,2*], Ze Wang[1], Tong Feng[3*], Jiandong Chen[1], Enkai Lin[1], An Xie[1]

[1]Key Laboratory of Functional Materials and Applications of Fujian Province, School of Materials Science and Engineering, Xiamen University of Technology, Xiamen 361024, PR China.

[2]National Key Laboratory of Electronic Thin Films and Integrated Devices, National Engineering Research, University of Electronic Science and Technology of China, Chengdu 610054, China.

[3]School of Mechanical Electrical and Information Engineering, Xiamen Institute of Technology, Xiamen, 361021, PR China.

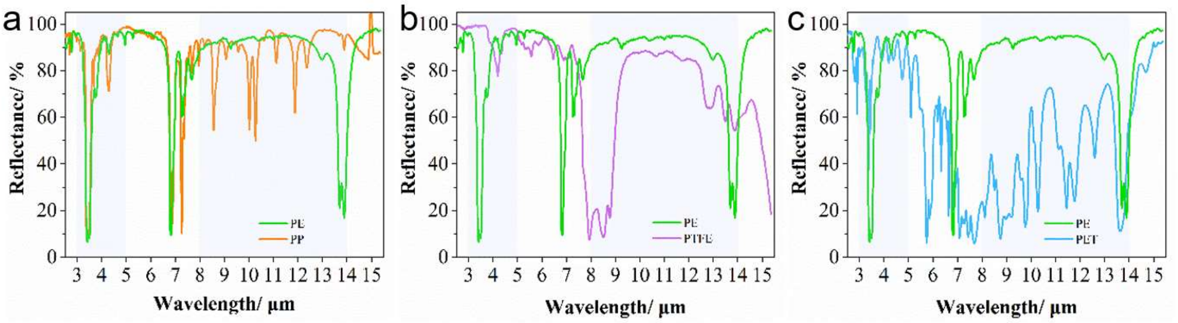

Figure S1 Comparison of infrared reflectance spectra of PE film with (a) PP, (b) PTFE, and (c) PET films.

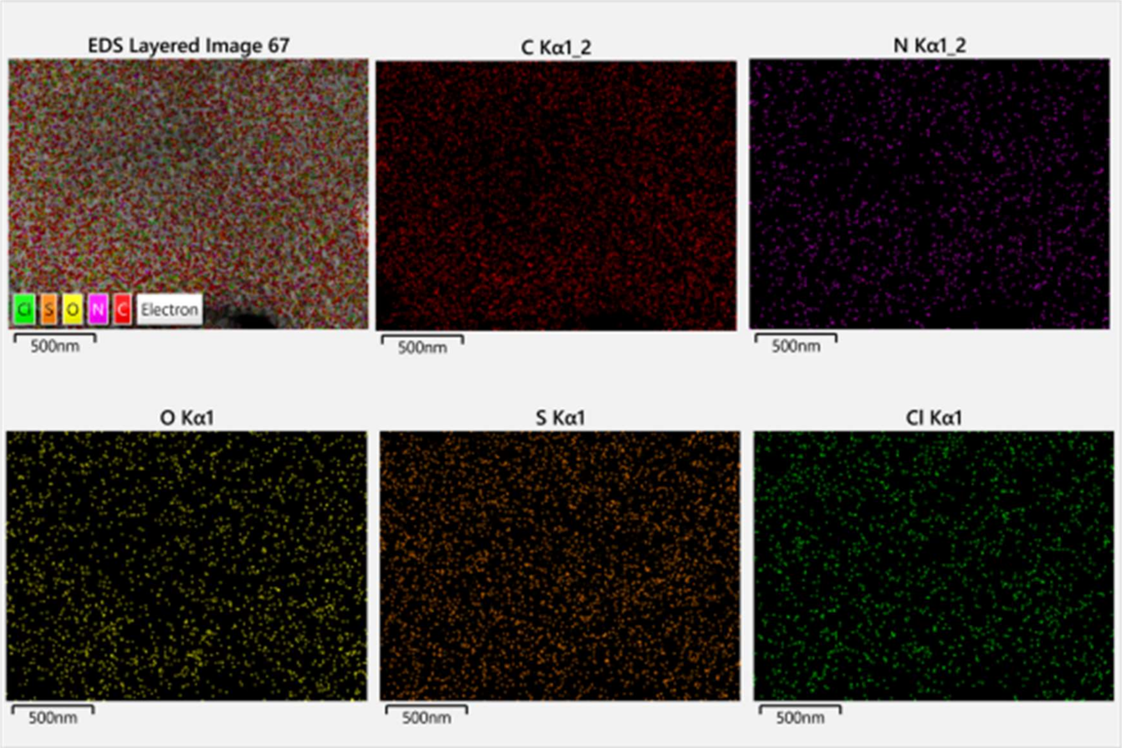

Figure S2. EDS mapping characterization of the SP(ANI-MA) film surface.

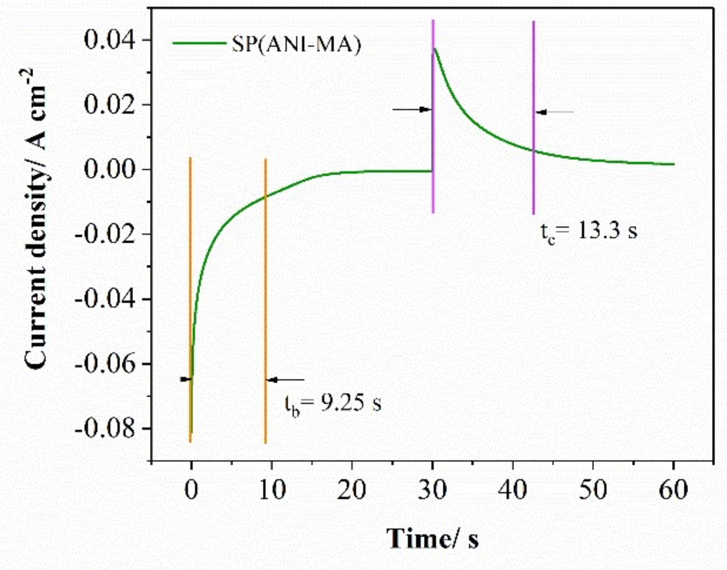

Figure S3. Response time characterization curves of the HWEC-ZIB device.
